# Penile erosions: an atypical initial manifestation of pemphigus vulgaris^؆^

**DOI:** 10.1016/j.abd.2025.501189

**Published:** 2025-08-09

**Authors:** Hugo J. Leme, José Ramos, António Magarreiro-Silva, Ana Isabel Gouveia, João Alves

**Affiliations:** Dermatology and Venereology Department, Hospital Garcia de Orta EPE, Almada, Portugal

Dear Editor,

Pemphigus represents a group of rare intraepidermal bullous autoimmune diseases.^1^ Pemphigus vulgaris (PV) is the most common subtype of pemphigus and is characterized by the presence of autoantibodies directed against epithelial adhesion proteins, Desmogleins (Dsg) 1 and 3, resulting in a loss of cell adhesion between keratinocytes.^2^, ^3^, ^4^ Penile involvement in PV is rarely documented, particularly as the initial manifestation of the disease.^1^, ^2^, ^5^ We report an atypical case of PV with onset in the penis and without involvement of other mucous membranes during the course of the disease.

A 52-year-old man presented with a 4-month history of painful penile erosions involving the glans and inner foreskin (Fig. 1). He had previously been diagnosed with candidiasis, balanoposthitis, and impetigo and was treated for those conditions without improvement. At the time of presentation to our department, the dermatosis had evolved with the appearance of lesions in other locations, namely flaccid blisters, erosions and adherent crusts in the inguinal region, trunk and scalp (Fig. 2) leading to the diagnostic hypothesis of PV. Histological examination of lesional skin was suggestive of PV (Fig. 3) and direct immunofluorescence in perilesional skin revealed intercellular deposits of IgG and C3 in the lower epidermis. Serum levels (detected by ELISA) of anti-Dsg-1 and anti-Dsg-3 IgG autoantibodies were >200 U/mL (normal <20) and 149,2 U/mL (normal <20), respectively, confirming the diagnosis. The patient was treated with oral prednisolone 100 mg/day (1 mg/kg body weight/day) along with azathioprine 150 mg/day. Due to the elevation of the liver enzyme levels, azathioprine was discontinued, and rituximab was started (two infusions of 1 g two weeks apart and an infusion of 1 g after 6-months as maintenance therapy). This resulted in a favorable clinical response with normalization of anti-Dsg 1 and 3 values, accompanied by a progressive reduction in prednisone dose over 6-months. The penile lesions took the longest time to heal and after 24-months of follow-up, the patient remains relapse-free.

**Fig. 1:**
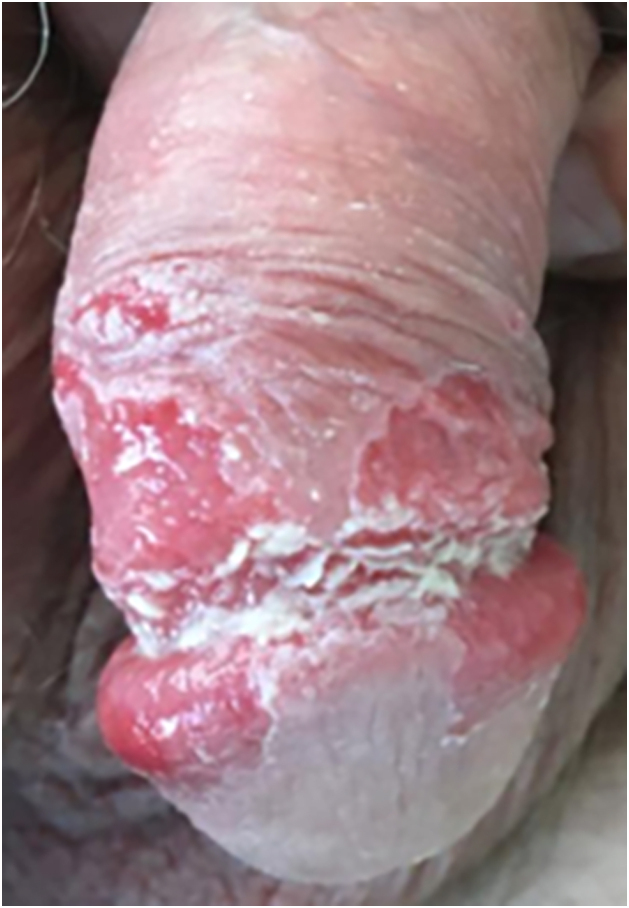
Penile erosions involving the glans and inner foreskin.

**Fig. 2:**
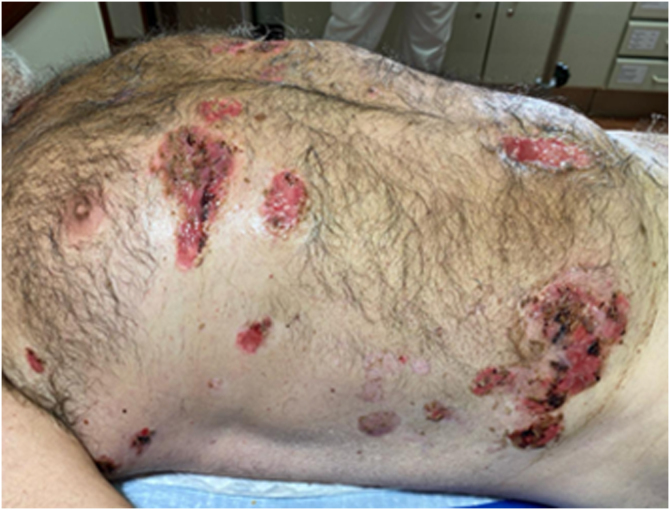
Flaccid blisters, erosions and adherent crusts in the trunk.

**Fig. 3:**
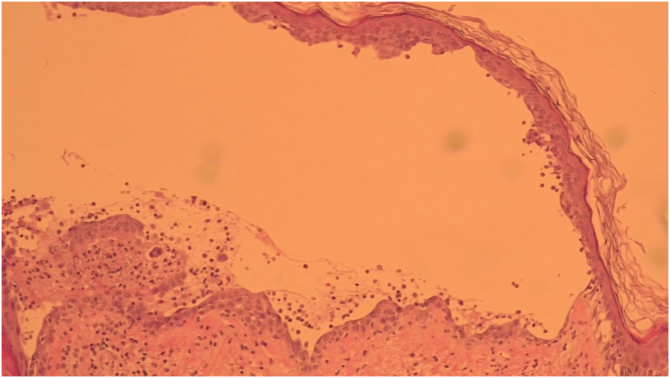
Suprabasal intraepidermal cleft with acantholytic cells, consistent with pemphigus vulgaris. Haematoxylin and eosin, original magnification 100×.

Although PV often manifests in the oral mucosa, involvement of other mucous membranes is less common and there are few cases reports in the literature of the penis being affected.^1^ In the present case, the initial presentation consisted of painful erosions on the penis. While subsequent involvement of other areas of the tegument was observed, the disease did not affect other mucous membranes. A study analyzing 12 patients with penile pemphigus^5^ found that 11 had oral involvement, 2 had esophageal involvement, and one patient each had involvement of the larynx, epiglottis, and conjunctiva. This highlights how uncommon is the involvement of the penis without the involvement of other mucous membranes.

In female patients, genital involvement is more frequent and is significantly associated with nasal involvement and genital symptoms.^6^, ^7^ Usually occurs when other sites are extensively affected.^7^ Involvement of the cervix, vagina and vulva has been described either concomitantly with other affected sites or when other involved sites were already in remission.^7^

In our patient the distinctive location in the genital region initially prompted the formulation of alternative diagnoses, resulting in the administration of unwarranted therapeutic interventions, a postponement in the diagnosis and a deterioration in the condition. Other differential diagnoses should include herpes simplex and drug-induced eruptions, both of which can present with similar erosion lesions. In addition, herpes infection has been associated with PV and may complicate therapeutic management.^8^

Clinicians should consider immunobullous diseases, such as bullous pemphigoid, mucous membrane pemphigoid, and PV as a cause for chronic mucosal lesions at genital sites. The presence of genital lesions may be associated with treatment resistance.^1^ Although rare, PV should be part of the differential diagnosis of painful erosions on the penis and, if in doubt, biopsies should be taken.

## ORCID ID

José Ramos: 0009-0007-5362-1141

António Magarreiro Silva: 0000-0002-0433-0352

Ana Isabel Gouveia: 0000-0001-7534-958X

João Alves: 0009-0004-2796-9986

## Financial support

None declared.

## Authors’ contributions

Hugo J. Leme: Preparation and writing of the manuscript; approval of the final version of the manuscript.

José Ramos: Approval of the final version of the manuscript; manuscript critical review.

António Magarreiro Silva: Approval of the final version of the manuscript; manuscript critical review.

Ana Isabel Gouveia: Approval of the final version of the manuscript; manuscript critical review.

João Alves: Approval of the final version of the manuscript; manuscript critical review.

## Conflicts of interest

None declared.
